# Meckel's diverticulum: A rare and missed cause of adult gastrointestinal bleed

**DOI:** 10.1002/ccr3.9000

**Published:** 2024-05-24

**Authors:** Rahul Ramakrishnan, Kevin Kuang, Vijay Rajput, Sachin Mohan

**Affiliations:** ^1^ Nova Southeastern University Dr. Kiran C. Patel College of Allopathic Medicine Fort Lauderdale Florida USA; ^2^ Department of Gastroenterology MedStar Health Oxon Hill Maryland USA

**Keywords:** balloon enteroscopy, Meckel's diverticulum, technetium, vitelline duct

## Abstract

Our report details a rare case of gastrointestinal bleeding in an adult male from Meckel's diverticulum. Diagnostic tests were negative except for technetium‐99m pertechnetate scintigraphy with SPECT/CT, highlighting importance of diverse modalities.

## 1 INTRODUCTION

Meckel's diverticulum (Md) arises from the incomplete elimination of the vitelline (omphalomesenteric) duct during the seventh week of gestation.^1^ In an embryo, the vitelline duct is a link between the developing gut and yolk sac to provide essential nutrition. Md was identified in 1598 by a German surgeon named Dr. Wilhelm Fabry. However, it was not until 1809 that Dr. Johann Friedrich Meckel, a German physician, provided a comprehensive description of the condition and was credited with the terminology of Md.^2^ This is a common asymptomatic congenital abnormality of the gastrointestinal tract. The common symptomatic presentation is abdominal pain with lower gastrointestinal bleeding (LGIB).

Md is present in around 2% of the population, 2% are symptomatic, common in children under 2 years old, two times more often in males, located approximately 2 feet from the ileocecal valve, measures around 2 inches long, and can have 2 potential epithelial types of gastric and pancreatic. These characteristics are often referred to as “rule of 2s.”^3^ We present a case of an adult male with LGIB from Md to highlight the importance of employing diverse diagnostic modalities to evaluate LGIB etiologies.

## 2 CASE HISTORY/EXAMINATION

A 23‐year‐old male with no prior medical history presented to the hospital after three episodes of bright red blood in his stool and 1 week of mild diffuse lower abdominal pain preceding the bleeding. He had no allergies, recent illnesses, nausea, vomiting, diarrhea, constipation, trauma, abdominal procedures, medications, travel, or alcohol use outside of occasional social events. He did not have any other risk factors or other complaints on review of symptoms.

The vital signs were stable without orthostasis. Physical examination noted a fully developed male with mild pallor and diffuse lower abdominal tenderness without hepatomegaly, asterixis, scleral icterus, or abdominal distension. The patient's hemoglobin level was 9.8 g/dL on admission (reference: 13.5–17.5 g/dL), which rapidly declined to 5.6 g/dL within 48 h. The patient encountered two to three episodes of hematochezia during hospitalization in the intensive care unit.

## 3 METHODS (DIFFERENTIAL DIAGNOSIS, INVESTIGATIONS, AND TREATMENT)

**FIGURE 1:**
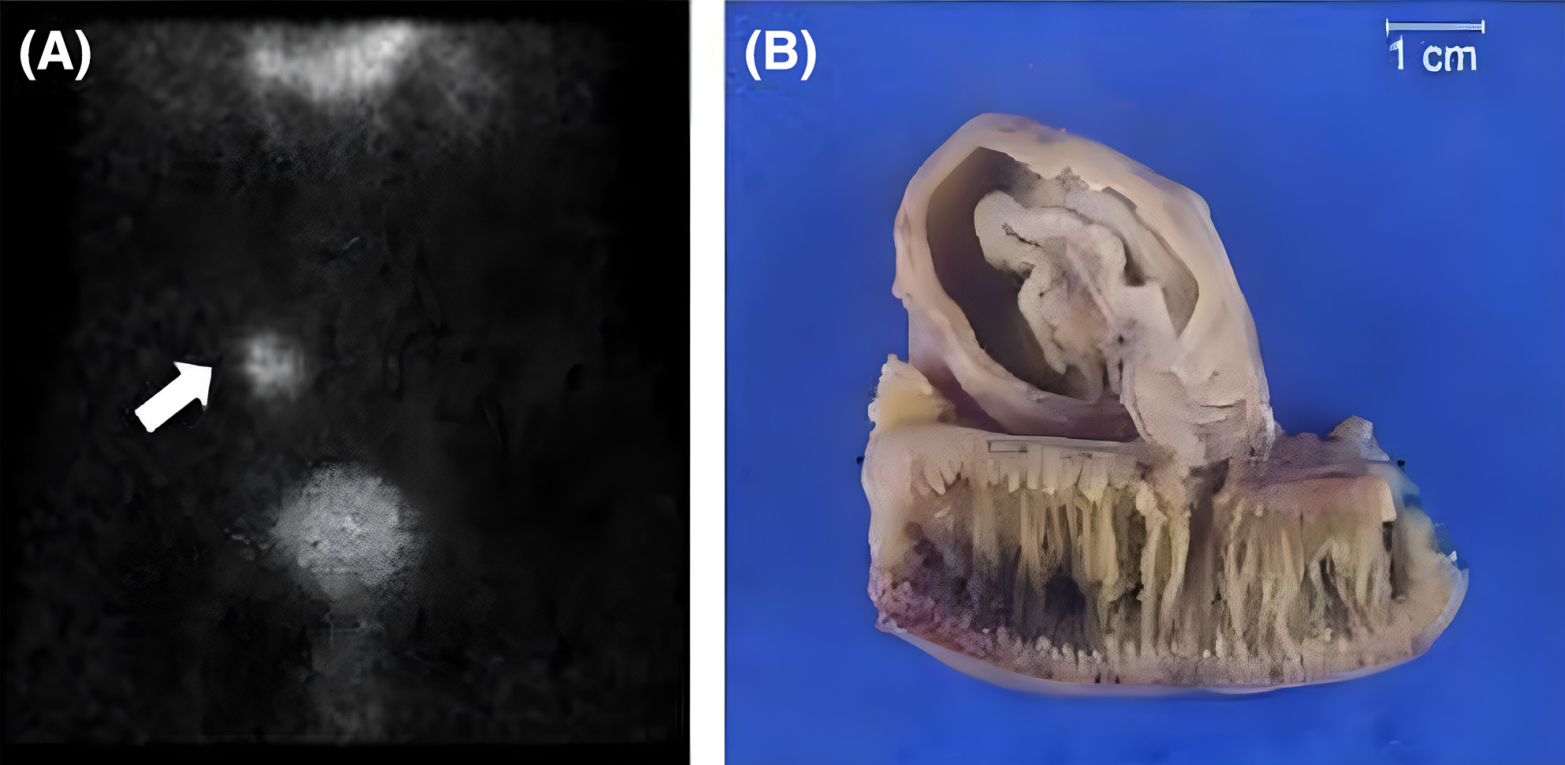
Technentium‐99m pertechnetate scintigraphy (A) indicating bleeding Meckel's diverticulum. Segmental resection of ileum with Meckel's diverticulum (B).

Patient required eight units of packed red blood cells (PRBC) and undergoing vigorous hydration. Conditions such as hemorrhoids, colonic polyps, and inflammatory bowel disease were considered as differential diagnoses based on patient's age and clinical presentation. He underwent diagnostic procedures, including upper endoscopy, colonoscopy, MR angiography, and video capsule studies. All the studies were nonconclusive in identifying the source of bleeding. A technetium‐99m pertechnetate scintigraphy, followed by SPECT/CT, was performed that revealed a bleeding Meckel's diverticulum at the distal ileum (Figure 1A, bold arrow). Subsequently, emergent laparoscopic resection of Md was performed. The histopathology was consistent for Md with hemorrhagic gastric mucosa epithelium lining (Figure 1B).

## 4 CONCLUSION AND RESULTS (OUTCOME AND FOLLOW‐UP)

At the time of discharge, the patient was clinically stable with a hemoglobin of 10 g/dL with no further bleeding or pain. He was discharged with stable hemoglobin levels and oral iron supplements.

## 5 DISCUSSION

In adult patients, symptomatic Md can mimic common GI conditions that range from hemorrhage, peritonitis, and diverticulitis to symptoms related to intestinal obstruction near the terminal ileum. Suspicion of Md in children necessitates technetium‐99m pertechnetate scintigraphy (Meckel scan), an imaging procedure that allows uptake by ectopic gastric mucosa. In contrast, adult patients with unrevealing diagnostic tests or hemodynamic instability may warrant an explorative surgery with subsequent surgical resection.^4^ Md must be in the differential diagnosis in adults with small intestinal bleeding without conclusive findings from an upper endoscopy, colonoscopy, MR angiography, and video capsule study.

Previous cases have documented instances of adult patients experiencing LGIB attributed to a bleeding Md. In 1990, Baumgartner et al.^5^ reported the case of a 24‐year‐old adult male presenting with diffuse abdominal tenderness and LGIB. Diagnostic tests yielded inconclusive results except for a positive finding on a Meckel's scan. Lin et al.^6^ described a similar case in 2002, involving a 24‐year‐old man with LGIB due to Md. Despite a history of occult GI bleeding spanning 5 years, results were negative from a Meckel's scan, colonoscopy, and small bowel enteroscopy completed 5 years earlier at a different hospital. In 2022, Rayan et al.^7^ reported on a 65‐year‐old adult male presenting with a 2‐day history of LGIB and abdominal tenderness attributed to Md. The patient arrived at the emergency department following a syncopal episode with trauma to the head. Subsequently, in the following year, Eddin et al.^8^ outlined the case of a 44‐year‐old adult male with LGIB due to Md. Initially, his hemoglobin level was 12.1 g/dL, which decreased to 9.4 g/dL after 12 h. In all four cases, similar to our own case report, patients underwent exploratory laparotomy with segmental small bowel resection to promptly address the LGIB. In contrast, Krstevski et al.^9^ reported in 2021 on a 33‐year‐old female with LGIB and abdominal tenderness due to a Md, whose symptoms were managed for 4 months using an H2 blocker and a proton pump inhibitor (PPI) before eventually undergoing laparoscopic surgery.

Diagnostic workup of a bleeding Md involves tools such as Meckel's scan, mesenteric arteriography, or capsule endoscopy. These techniques can have a low diagnostic yield. For instance, capsule endoscopy, Meckel's scan, and mesenteric arteriography can have a diagnostic yield of 7.7%, 21.4%, and 19%–92%, respectively.^9^, ^10^, ^11^ Meckel's scan is considered the gold standard for diagnosing both bleeding and other symptomatic Mds. While Meckel's scan has a sensitivity ranging from 85% to 97% in pediatric patients, it has a lower sensitivity and lower positive predictive value of 55%–62% in adults.^6^, ^12^ Potential for false‐negative results in Meckel's scan arises from the presence of ectopic pancreatic tissue and is further influenced by limitations associated with intestinal obstructions. Balloon‐assisted enteroscopy (BAE) is not commonly utilized for Md evaluation, yet BAE is an effective technique when compared to other diagnostic modalities. In recent years, there has been an increase in the utilization of BAE for addressing small intestinal bleeding such as in Crohn's disease, foreign body retrieval, tumors, and lesions associated with malignant tumors and inflammatory bowel disease.^13^, ^14^, ^15^ BAE involves the use of a flexible tube with an inflatable balloon that is inserted through the mouth or anus. A controlled back‐and‐forth movement allows visualization of the small intestine, and particularly, the diverticular ostium in Md.^16^ Two types of BAE are single balloon enteroscopy (SBE) or double balloon enteroscopy (DBE). Dr. Hironori Yamamoto in 2001 designed DBE to address small bowel conditions such as Crohn's disease, while SBE was developed in 2007.^17^

In one retrospective review of 35 symptomatic Korean adults with small bowel bleeding in Md and various diagnostic modalities, the BAE modality had the highest diagnostic accuracy (85%) in contrast to Meckel's scan (21.4%), angiography (0%), and capsule endoscopy (35.7%).^4^ At present, BAE is available in tertiary care centers across the United States and is not easily available in nonacademic health care facilities. The authors of this paper anticipate BAE's use to expand in future as a diagnostic tool preoperatively for LGIB and bleeding Md cases in adult population. BAE can not only allow patients to avoid additional diagnostic procedures but also curtail costs. Endoscopic resection using BAE can be curative in management of Md, but due to lack of BAE availability, Meckel's scan followed by targeted surgical resection remains the mainstay of Md management.

Overall, our case emphasizes the importance of considering Md in the differential diagnosis of LGIB in adults for early recognition and intervention. The utilization of a broad differential diagnosis list and numerous diagnostic modalities is imperative when evaluating LGIB in adult patients.

## AUTHOR CONTRIBUTIONS

**Rahul Ramakrishnan:** Writing – original draft. **Kevin Kuang:** Writing – original draft. **Vijay Rajput:** Supervision; writing – review and editing. **Sachin Mohan:** Conceptualization; supervision; writing – review and editing.

## FUNDING INFORMATION

None to declare.

## CONFLICT OF INTEREST STATEMENT

The authors have no conflict of interest to declare.

## CONSENT

Written informed consent was obtained from the patient to publish this report in accordance with the journal's patient consent policy.

## Data Availability

Findings of this study are accessible upon request from the corresponding author.
